# Breast Cancer Detection Using Automated Segmentation and Genetic Algorithms

**DOI:** 10.3390/diagnostics12123099

**Published:** 2022-12-08

**Authors:** María de la Luz Escobar, José I. De la Rosa, Carlos E. Galván-Tejada, Jorge I. Galvan-Tejada, Hamurabi Gamboa-Rosales, Daniel de la Rosa Gomez, Huitzilopoztli Luna-García, José M. Celaya-Padilla

**Affiliations:** Unidad Académica de Ingeniería Eléctrica, Universidad Autónoma de Zacatecas, Jardín Juarez 147, Centro, Zacatecas 98000, Mexico

**Keywords:** cancer, PyRadiomics System, genetic algorithm

## Abstract

Breast cancer is the most common cancer among women worldwide, after lung cancer. However, early detection of breast cancer can help to reduce death rates in breast cancer patients and also prevent cancer from spreading to other parts of the body. This work proposes a new method to design a bio-marker integrating Bayesian predictive models, pyRadiomics System and genetic algorithms to classify the benign and malignant lesions. The method allows one to evaluate two types of images: The radiologist-segmented lesion, and a novel automated breast cancer detection by the analysis of the whole breast. The results demonstrate only a difference of 12% of effectiveness for the cases of calcification between the radiologist generated segmentation and the automatic whole breast analysis, and a 25% of difference between the lesion and the breast for the cases of masses. In addition, our approach was compared against other proposed methods in the literature, providing an AUC = 0.86 for the analysis of images with lesions in breast calcification, and AUC = 0.96 for masses.

## 1. Introduction

Artificial Intelligence may help in the detection and diagnosis of any disease. Moreover, in cancer diseases, early detection is essential to prevent the spread of cancer in the body, resulting in the patient’s death. Breast cancer is one of the most aggressive types of cancer, and is responsible for almost 685,000 deaths in females worldwide [1]. In Mexico, breast cancer has increased between the years from 2013 to 2016, with 24,695 women deaths [2]. Thus, an early diagnosis is critical for breast cancer survival [3]. Screening mammography is the preferred early detection strategy for reducing breast cancer mortality [4]. Mammography screening has had a positive impact in about 35% of breast cancer detection [5]. On the other hand, the CAD’s systems try to emulate the process realized by the radiologist for detecting the cancer. Detection of early breast cancer signals is a routine and repetitive procedure. From the typical radiologist breast cancer subjects, only 0.4% of the cases are malignant [6].

Aiming to reduce the load of work for the radiologist, computer-aided detection (CAD) systems are designed to assess the radiologist, as a second opinion, and it may aid in the correct interpretation of suspicious findings [7,8,9,10]. This process is not a trivial task due to the heterogeneity of abnormalities and the darkening under dense masses, making it difficult to identify a possible breast cancer. Mammography analysis helps to analyze the internal structure of the breast, with the aim of studying the tissues and injuries such as nodules, classifications, asymmetries in breast density and distortion of the architecture of the breast [11,12,13,14]. The features seek to provide information about the shape, contour, density, and perimeter and correspond to the input of an artificial intelligence system to classify the lesion into benign or cancer [15]. The relationship between breast lesion analysis and morphological description has been widely investigated [16]. Other research has been focused on extracting features with the viogram function as a texture feature descriptor. Other features are extracted using a new hybrid of scheme of texture, co-occurrence matrix and geometric features with a neural network [17].

On the other hand, pyRadiomics is a tool for medical imaging that allows feature extraction. The pyRadiomics toolkit was used for tissue characterization investigated by Granzier [18]. Gao et al. have performed a similar series of experiments using the pyRadiomics platform for prediction of the auxiliary lymph node tumor burden in breast cancer patients [19]. Vamvakas investigated the utility of boosting ensemble classification methods for increasing the diagnostic in differentiating benign and malignant breast lesions [20]. The fist idea is to reduce the quantity of features, which give the benefit to obtain low computational costs. For example, in the investigation proposed by Galván-Tejada et al. [21]. Galvan proposed a multivariate model that classifies the lesion into benign or malignant tumors using a genetic algorithm that analyze the morphological characteristics of the lesions to obtain an optimal classification. Genetic algorithms as an optimization tool for feature selection models have been revealed as an efficient technique using a computer-assisted diagnosis, so this approach will be also used in this investigation [22,23,24].

Refs. [25,26]: Reports demonstrate that ML models allow one to reduce false positives when classifying lesions, using optimization techniques on images. Moreover, some cross-sectional studies suggest an association between fatty and fatty-glandular for the analysis of mammography using a set of micro calcification features. Other research, which is based on texture description, spectral clustering, and Support Vector Machine (SVM) for the detection of breast masses [27], also aims to obtain more informative features. Other multivariate analysis approaches have demonstrated that prognostic information and predictive factors can be obtained to identify breast cancer in its early stages [28]. Among the different techniques of digital image processing and pattern recognition that have been applied in breast cancer, the use of mutual information and a greedy selection are used for this diagnosis when the information is uniformly distributed [29]. The feature selection for classifying benign and malignant lesions could also be made by using standard classification algorithms such as: K-nearest neighbors (KNN), decision trees, and naive Bayes [30]. On the other hand, Haralick et al. [31] introduced for the time the concept of Co-Occurrence Matrix (GLCM) for the analysis of texture patterns and their spatial classification. These relationships are specified in the built-in co-occurrence matrix for breast texture classification, since in recent works, the co-occurrence matrix for texture classification in breast images has been incorporated [32]; this concept will be also considered in our proposition.

On the other hand, Tsochatzidis et al. investigated the performance of multiple networks for breast cancer diagnosis from mammograms with mass lesions [33]. The incorporation of a margin-specific content-based image retrieval approach into a computer-aided diagnosis scheme of mammographic masses is investigated for the same authors in [34]. Andrik proposed a method, which is based on AlexNet with some modifications and has been adapted to our classification problem [35]. A deep ensemble transfer learning and neural network classifier for automatic feature extraction and classification was proposed by Aurora [36]. It should be mentioned that the authors also work with the CBIS-DDSM images. Furthermore, three data sets investigated a CAD system based on deep Convolutional Neural Networks (CNN) for classifying mammography mass lesions [37].

Feature analysis plays an important role in developing a specialized software for extracting the key features and building a robust classification scheme; numerous experiments have been implemented in the pyRadiomic system [38]. In this work, a predictive model was implemented for the detection of lesions in calcification to classify between the benign and malignant breast. The focus of the work is to speed up the diagnosis of breast cancer using the genetic algorithm and PyRadiomics System. Subsequently, the diagnosis can be confirmed by radiology through workflow.

The remainder of the paper is organized according to the following sections: The first section of this paper will examine other investigations in the literature Section 1. Section 2 describes the materials and methods. The experimental design is presented in Section 3. The results and discussion are presented in the Section 4 and Section 5. Finally, the last gives a conclusion in Section 6.

## 2. Materials and Methods

**Figure 1:**
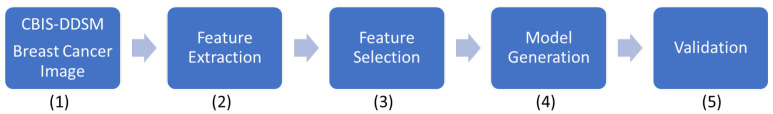
Block diagram of the proposed methodology.

The proposed methodology is presented in the block diagram on Figure 1. In the first stage (1), the mammography data set used in this methodology is described and it corresponds to the input data. The feature extraction method is based on a set of data extracted on the image using the PyRadiomics System (2). The process to reducing the number of features is proposed in stage (3). The classification between benign or malign is described in the stage (4). Finally, in stage, the validation of the model was realized through proof measures of efficacy, to correctly predict two models: calcification and masses for benign and malignant lesions (5). All previous stages are detailed in the following subsections.

### 2.1. Data Acquisition and Segmentation

#### 2.1.1. Data Description

**Figure 2:**
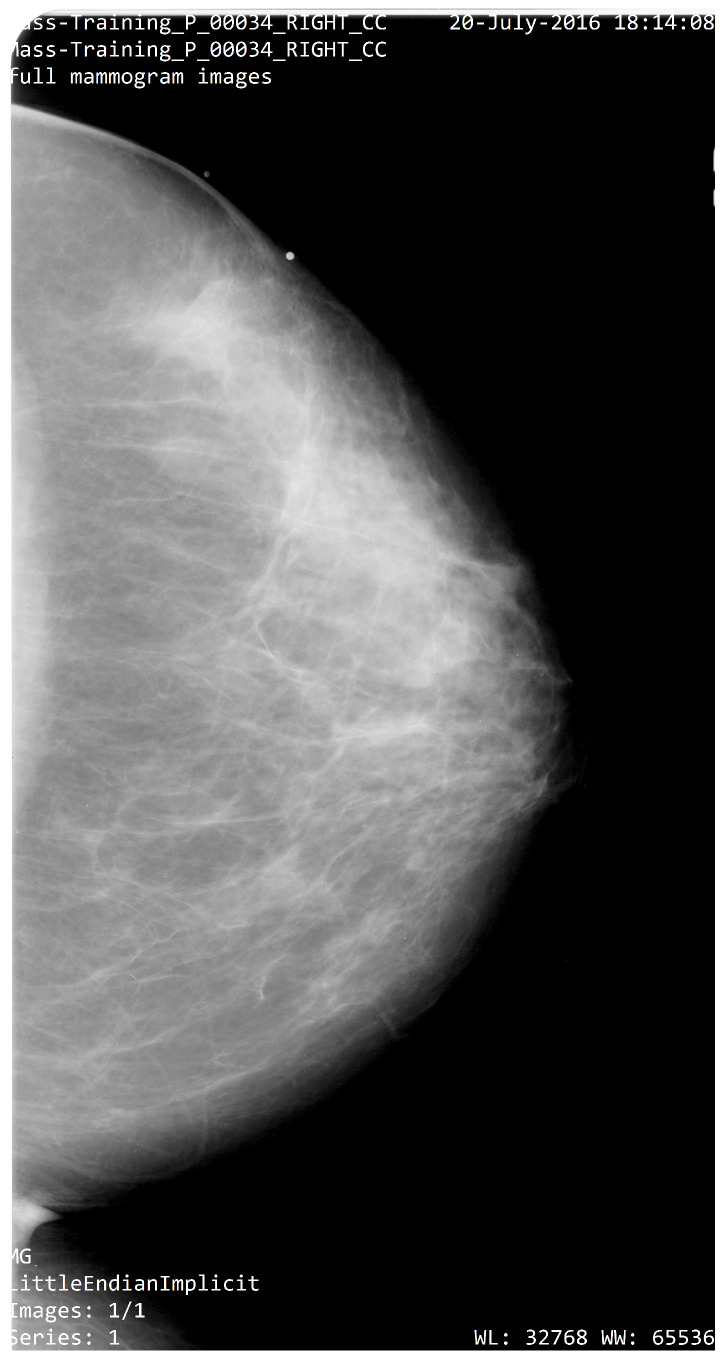
Original mammogram (CC) obtained from CBIS-DDSM data-sets.

The Digital Data base for Screening Mammography (DDSM) is a data base collection of 2620 study cases at the University of South Florida [39]. This data set includes two images of each breast, patient information and an image information system. Recently, another data base was extracted and standardized to test scientific methodologies, and evaluate their performance [40]. This CBIS-DDSM (Curated Breast Imaging Subset of DDSM) is a data subset of the original DDSM database. The digital mammography was decompressed and converted to a Digital Imaging and Communications in Medicine (DICOM) format (as shown in Figure 2). The data set imaging contains the left and right of the craniocaudal view (CC) and medial lateral oblique view (MLO) of the breast images for each patient. The data set also includes descriptions of the location of the breast lesion on calcifications and masses, bounding boxes, and pathology diagnosis for training test images. In order to complete the first stage, it is also necessary to provide a region-of-interest (ROI) segmentation on the breast lesion. Abnormalities were cropped by determining the bounding rectangle of the abnormality with respect to its ROI. The data set contains 753 calcification cases and 891 mass cases. There are Breast Imaging Reporting and Data System (BI-RADS) descriptors for mass shape, mass margin, calcification type, calcification distribution, and breast density. Table 1 shows some characteristics of the CBI-DDSM.

#### 2.1.2. ROI Segmentation

**Figure 3:**
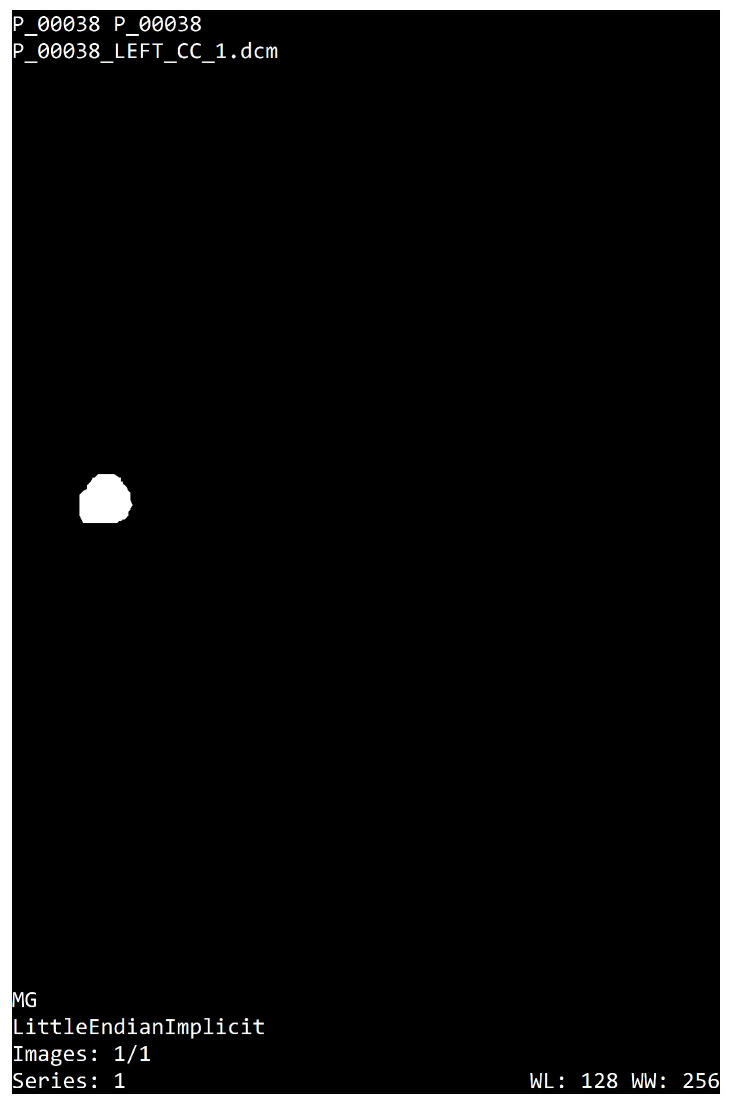
ROI segmentation.

The data base CBIS-DDSM includes a mask of the region of interest which represents the location of the lesion and its pathology. DDSM outlines provide only a general location and not a precise mass boundary. Subsequently, a segmentation algorithm was proposed to provide the exact delineation of the mass from the surrounding tissue. This segmentation was realized only for masses and not for calcifications. All images in the DDSM were provided from several different scanners at different institutions. We used the image with the same scanner to avoid contrast problems. The data set thus contains a binary mask segmented for the radiologist where the value 255 (white color) represent the segmented lesion and 0 (black color) correspond to the background information, as shown in the Figure 3.

#### 2.1.3. Breast Regions

The mammography analysis by a specialist allows for the diagnosis of cancer. However, the long time period of the work flow performed by the radiologist allows the disease to progress, or to spread it throughout the body. In this research, It proposes a methodology based on the analysis of the breast, and with the help of artificial intelligence algorithms, predicts whether there is a benign or malignant lesion in the breast. This would allow the radiologist to have a rapid diagnostic response. Reducing delays in diagnosis or performing repetitive tasks is the main goal of the proposed research.

In this subsection, It will perform the segmentation of the breast for subsequently introducing it to the PyRadiomics tool and then changing it to the feature extraction (second stage). Firstly, the segmentation is focused on the characterization of the background image to create a binary image to be used as segmentation mask, then the segmentation technique validates a pixel group based on a global threshold. The method consists in finding the target region that can be an approximation of the whole area of the breast. This technique also allows one to find a global threshold. Therefore, the selected targets have a threshold value higher than 50 bits. The biggest area in the image has been chosen, which represents the breast or ROI, as shown in Equation (1). As a result of the image segmentation process, one mask has been generated with normalized intensity from 0 to 1 (where 1 represent information into the image and zero corresponds to the background).

(1) $$\begin{array}{c} \max\left(ROI\right)=\left\{\begin{array}{cc} P(I{\left(R_{i}\right)}_{i,j})>50, & 1,\\ \mathrm{otherwise}, & 0. \end{array}\right. \end{array}$$

(2) $$\begin{array}{c} X_{i,j}=M_{i,j}\&I_{i,j}. \end{array}$$

The Equation (2) defines $I_{i,j}$ as the original image. Let $I_{i,j}$ be the set of integer values ranging from 0 to 255, then a digital image in DICOM format, where i,j is the set of pixels in the original image, whose elements are pairs of natural integer numbers, whose components correspond to the relative position of each pixel in the image. The *i* values represent the raw and *j* the columns. *M* represents the segmentation mask normalized between [0,1]. Finally, & represents the binary operation, and $X_{i,j}$ is the resulting image.

**Figure 4:**
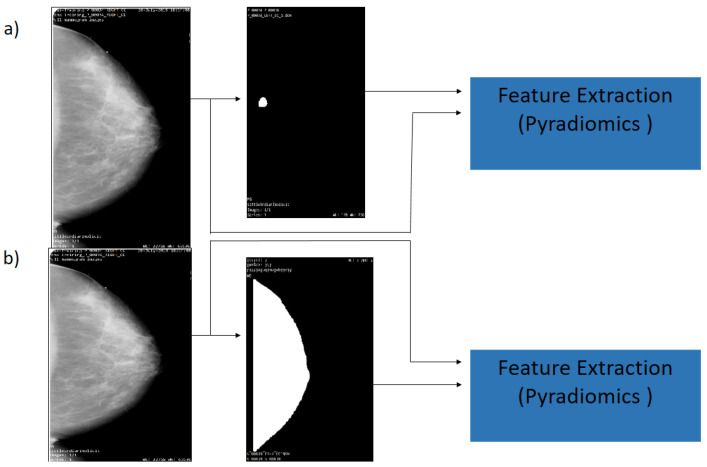
Flowchart of the mammography segmentation. (**a**) Original image and ROI Segmentation (**b**) Original image and whole breast.

In this research, the analysis is focused on the lesion segmentation and over the region of the breast. Then, the previous results of the segmentation process provide the mask and the ROI breast, and the CBIS-DDSM data set provide mask lesions, which indicate the region where the lesion is located. A medical image and segmentation mask with format DICOM are the input data for PyRadiomics System (see Figure 4). On the input, it takes an initial input of binary masks, which are defined as follows: The mask is realized by the experience of the radiologist to segment the lesion, as shown in Figure 4a). Subsequently, in the Figure 4b),the breast segmentation is demonstrated, as realized by the authors.

### 2.2. Feature Extraction

**Figure 5:**
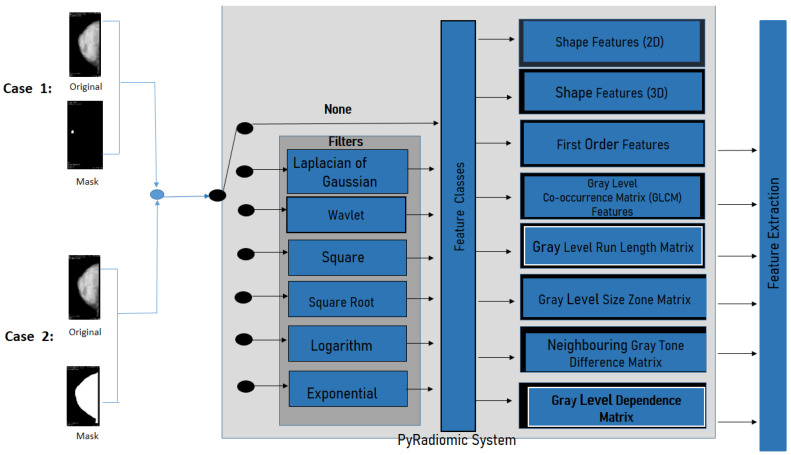
PyRadiomics System configuration for this work.

The PyRadiomics System is a specialized package for radiomics feature extraction from medical imaging [41]. The extracted radiomics characteristics have been validated by researchers trying to establish a standard framework into the Imaging Biomarker Standardization Initiative document (IBSI) [38]. The installation of the PyRadiomics System has been made by a compilation of source archives in python version 3.5(64−bits). Then, one can proceed to extract features from medical images, conduct 2D and 3D segmentation, and the binary mask (as shown in Figure 5). This system allows one to obtain 8-based-classes into a morphological context; it is also possible to obtain texture features, and the relationships between pixels or voxels. Further morphology features, first order statics, can also be extracted.

Feature Extraction is based on texture analysis and the geometry of the ROI; some of these characteristics are demonstrated in Table 2.

Furthermore, PyRadiomics developed an optional platform, which uses built-in filters as Laplacian of Gaussian, Wavelet, Square, Square Root, Logarithm, Exponential, Gradient, and Local Binary Patterns 2D or 3D. For this investigation, the geometric feature was eliminated, and filter was chosen (as shown in the filtering stage in Figure 5).

### 2.3. Feature Selection

#### 2.3.1. Removing Features with Zero Variance

The PyRadiomics System generates a high dimensional space of features, as shown in the feature matrix of Equation (3). With the aim to reduce the size of features, it was realized as removing process, and thus the feature selection (third stage) consists in two steps. In the first step, only those columns with zero variance were removed, subsequently, in the second step, the feature selection was carried out using genetic algorithms and considering the remaining elements of the feature matrix. The extracted data set is represented by the following feature matrix (3).

(3) $$\begin{array}{c} \left[\begin{array}{cccccc} X_{1,1} & X_{1,2} & X_{1,3} & X_{1,4} & \ldots & X_{1,n}\\ X_{2,1} & X_{2,2} & X_{2,3} & X_{2,4} & \ldots & X_{2,n}\\ X_{3,1} & X_{3,2} & X_{3,3} & X_{3,4} & \ldots & X_{3,n}\\ \vdots & \vdots & \vdots & \vdots & \; & \vdots \\ X_{m,1} & X_{m,2} & X_{m,3} & X_{m,4} & \ldots & X_{m,n} \end{array}\right] \end{array}$$

Let us write this matrix as an ordered set of column vectors, where the matrix $X_{j,i}$ represents the *j*-th row of features and the *i*-th sample vector. In order to compute the covariance matrix $R_{x,y}=E\left(x_{pj},x_{qj}\right)$, where $Var[X_{n,m}]=0$, and where $D=\mathrm{diag}\{\sigma_{1}^{2},\sigma_{2}^{2},\sigma_{3}^{2},\ldots ,\sigma_{m}^{2}\}$ is the diagonal variance, which leads to a new covariance matrix $S_{i,i}$ when $\sigma_{k}^{2}\neq 0$.

#### 2.3.2. Data Whitening

In the third stage, it is important to center the extracted data set $x_{k}$; this process is well known as data whitening, and it considers that data must have a zero mean and variance one.

(4) $$\begin{array}{c} s_{i}=\frac{M(S)}{std(S)}. \end{array}$$

The Equation (4) is defined as the test data $s_{i}$, where M(S) is the arithmetic average, and std(S) is the standard deviation of $S_{i,i}$ giving, as result, the standard normalization.

### 2.4. Feature Selection Based in a Genetic Algorithm

Feature selection is a method implemented to select the best features and then ensure a better classification. One of these feature selection methods are the so-called Genetic Algorithms.

Genetic algorithms (GA) are optimization techniques based on natural selection where certain genetic information is transmitted from one generation to the next. This process of evolution modifies a set of solutions (chromosomes) by selecting individuals with the best features (gens). The selection is performed using a “fitness” function. The selected individuals (parents) will produce the next generation (children). This evolutionary process will eventually achieve an optimal solution.

It implement the genetic algorithm using Galgo [23]. This software is an object-oriented programming (OOP) implementation in R. Further, it includes the code to develop models using Random Forest [28,45].

**Figure 6:**
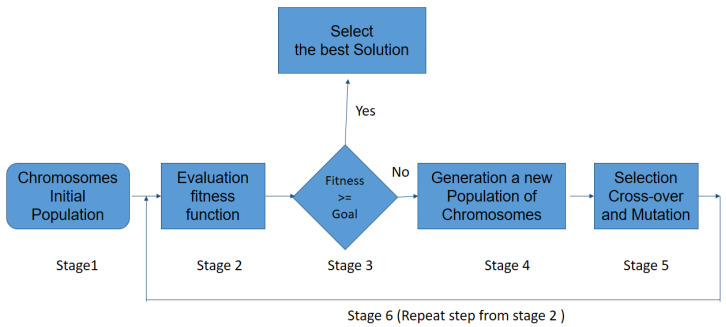
Schematic representation of the GA procedure.

**Figure 7:**
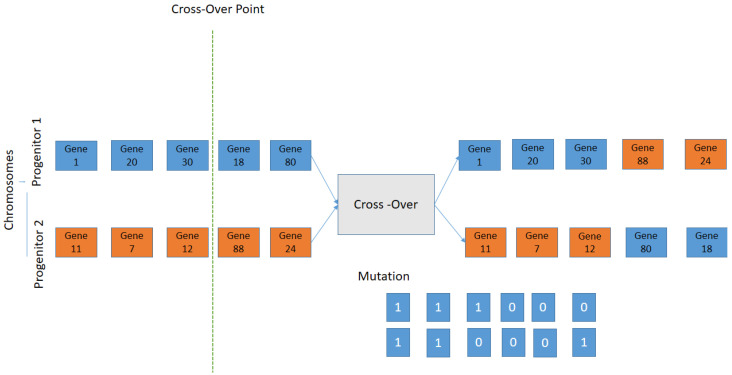
Schematic representation of the Crossover; the selection criteria is used by exchanging the genes of parents from one generation to the next.

The stages of the protocol used by Galgo are described below (Figure 6):

1 An initial population consisting of possible solutions to the problem, also called individuals, is randomly generated. This variable is called chromosomes $S_{i}$, with i=1,…,N.
2 Calculate the fitness function *F* of each chromosome in the population, and evaluated for the ability to predict the group membership of sample (pϵS).
3 If the chromosome is selected and the procedure stops; otherwise, the procedure (stage 1) continues to stage.
4 Cross-Over: the two selected individuals generate new offspring with a higher fitness score (see Figure 7).
5 The mutation process is to add a new individual to the new population. The new gene generated by the mutation is aggregated at the chromosomes.
6 The process is repeated from stage 2 until an accurate chromosome is obtained [46].

### 2.5. Model Generation

For the estimation of the parameters, the maximum-likelihood framework has been chosen, using the logistic regression model (see Equation (5)). Where *Y* is the variable to predict, and $s_{1},\ldots ,s_{n}$ are the *n* predictor variables, the logistic regression paradigm is expressed as follows:

(5) $$\begin{array}{c} P_{t}\left(\theta ,Y=1|s_{n}\right)=\frac{e^{\theta_{0}+\theta_{1}\;s_{1}+\cdots +\theta_{p}s_{p}}}{1-e^{\theta_{0}+\theta_{1}\;s_{1}+\cdots +\theta_{p}s_{p}}}. \end{array}$$

In the previews Equation (5), *Y* determines the likelihood of malignant lesion, with an interval [0,1]; it uses a threshold equal to 0.5. Moreover, a Gaussian Distribution of data is assumed, with zero mean and variance $\sigma^{2}$ (see Equation (6))

(6) $$\begin{array}{c} y_{i}=P_{t}\left(s_{i},\theta \right). \end{array}$$

where θ evaluates the accuracy prediction of the model.

### 2.6. Validation

**Figure 8:**
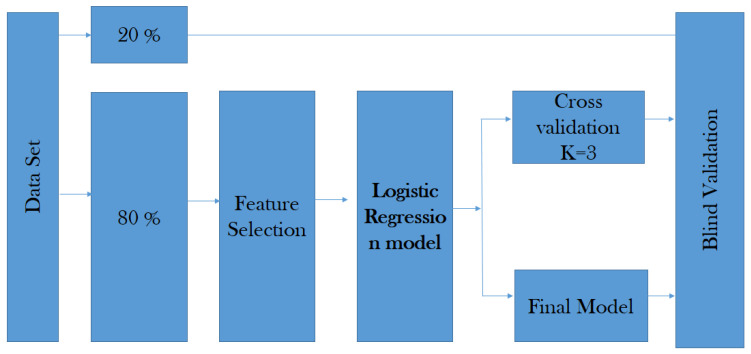
Proposed validation strategy.

The cross-validation is the technique used to evaluate the results of the predictive model by partitioning between the data into training and test sub-sets (see Figure 8).

The data is randomly mixed, forming a number of *k* data. The model uses *K* folds for each iteration to test the model, and the remaining *K* data set to train the model. The fold helps to test the data set and the other one supports the training process (see Equation (7)).

(7) $$\begin{array}{c} f=\frac{1}{N}\sum_{i=1}^{N}f_{i}. \end{array}$$

where *f* represents *K*-Fold Cross Validation, and *N* is the fold’s number of the data set into sets, training, testing, and validation (in our case, N=3).

The validation of the model is carried out from prediction measures, such as: Area under the curve, predictive measures over the classifier with the aim to distinguish between classes, and specificity and sensitivity are also used to calculate the positive and negative predictive values. Finally, accuracy provides the percentage of correct predictions for the prediction models.

## 3. Experimental Setup

In this research, some independent studies were used to explore between two types of breast lesion, benign or malignant, in images of calcification and mass. Left or right breast images with suspicious regions were only selected in the proposed experiments; a total of 400 left and right breast mammograms were used with the CC projection. For the calcification, the first sub-set (CS1) was obtained by using only the data contained inside the ROI segmentation provided by the radiologist; then, for the second classification sub-set (CS2), the whole breast segmentation was obtained. The same process was also used for the both sub-sets of the mass data set (MS1, MS2) (as shown in Figure 4).

The segmentation process is used to eliminate artifacts and labels from the mammogram image, and to select the breast ROI. A threshold value was used to extract the binary mask. Moreover, some morphological operations were applied for the segmentation mask to finally obtain the region of interest of the breast. The process feature extraction on images was realized using the PyRadiomics System. The PyRadiomics required the image and the mask input; for these experimental results, the cases CS1 and MS1 were used for the mask provided by the radiologist. On the other hand, in the MS1 and MS2 cases, the mask breast segmentation was used.

Once the mammography features were extracted by PyRadiomics, 141 features were selected with the basis of texture information from the lesion and from the breast segmentation, and the 21 shape descriptors were removed. Gray Level Co-occurrence Matrix, Gray level Run Length Matrix, Gray level Size Zone Matrix, Neighbouring Gray Tone Difference Matrix, and Gray level feature was selected for this experiment.

Then, in order to select the best features to construct a robust model, a feature selection process was implemented into two stages; in the first one, the no-variance features were removed, then on the second stage, a genetic algorithm (GALGO) [23] was used to search for the best combination of features that correctly classify the samples.

Then, a validation was carried out by means of cross-validation for each CS1, CS2, MS1 and MS2 sub-sets. A cross-validation with a k=3 strategy was used, then a series of metrics were computed in order to assess the performance of the models on unseen data for this, and the AUC, sensitivity, specificity, and accuracy were calculated. Firstly, we shuffled the data set to make up *k* different sub-sets for the training and test phases.

## 4. Results

In this section, some results are obtained considering four sub-set cases of images with mass and calcification. This process allows one to read DICOM images converted into a binary image from a gray level. The experiment consisted of 400 images; two types of malignant and benign lesions between right or left images are considered for all cases, ROI segmentation is provided by the radiologist and breast segmentation is obtained according to the proposed methodology.

**Figure 9:**
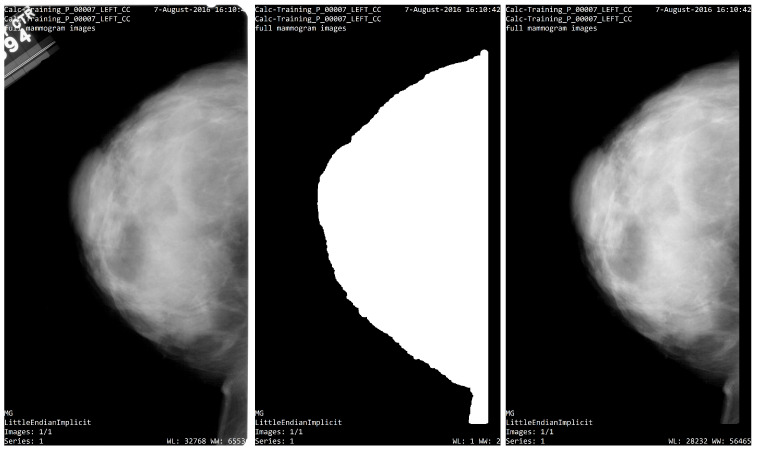
Segmentation process. (**left**) Image from CBIS_DDSM; (**center**) segmentation mask; (**right**) ROI segmentation.

The breast segmentation process was based on contour detection; first, the algorithm finds all the objects inside the input image, then the area containing such objects is computed, next, the biggest area is selected as a candidate for the breast organ. Once the breast organ is selected, all other objects are eliminated leaving only the breast organ. Nevertheless, several of the input images have noise or unwanted tissue on the frame boundary, and to eliminate such artifacts, 5% of the edge of the image is removed, creating a segmentation mask that only contains breast tissue; the Figure 9 shows an example of this process.

**Figure 10:**
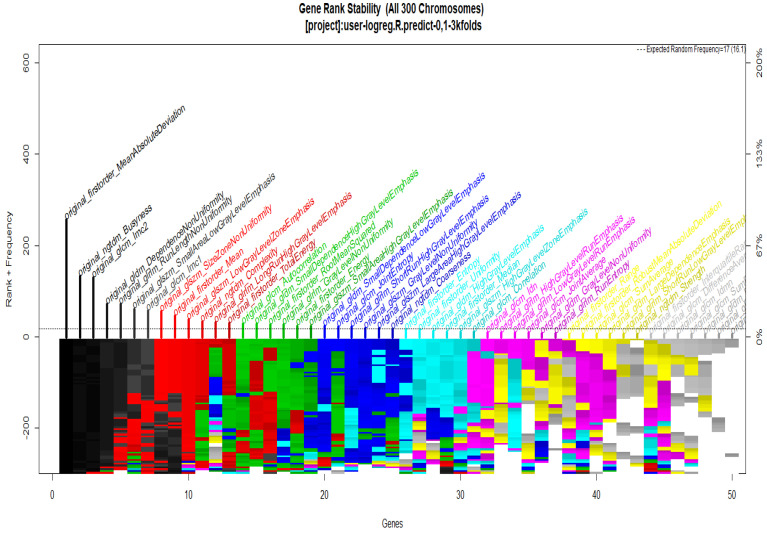
Gene Rank Stability graph with calcification model by radiologist segmentation; the gene rank shows the stability of the top-ranked 50 variables. The horizontal axis shows the genes ordered by rank, and the vertical axis shows the gene frequency. The bottom color—coded rank represents the genetic stability where features with no change in the color represent a stable feature; changing color features represent unstable features (i.e., not always contributing to the performance).

**Figure 11:**
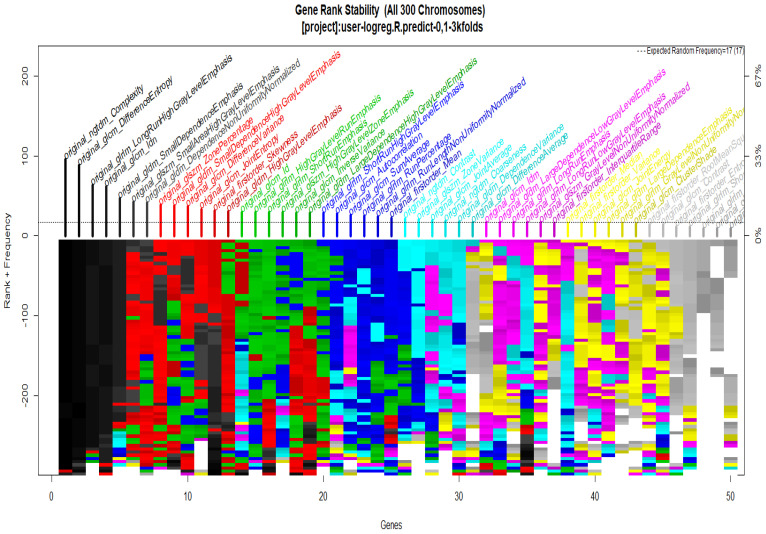
Gene Rank Stability graph with calcification model by radiologist segmentation; the gene rank shows the stability of the top-ranked 50 variables. The horizontal axis shows the genes ordered by rank, and the vertical axis shows the gene frequency. The bottom color—coded rank represents the genetic stability where features with no change in color represent a stable feature; changing color features represent unstable features.

**Figure 12:**
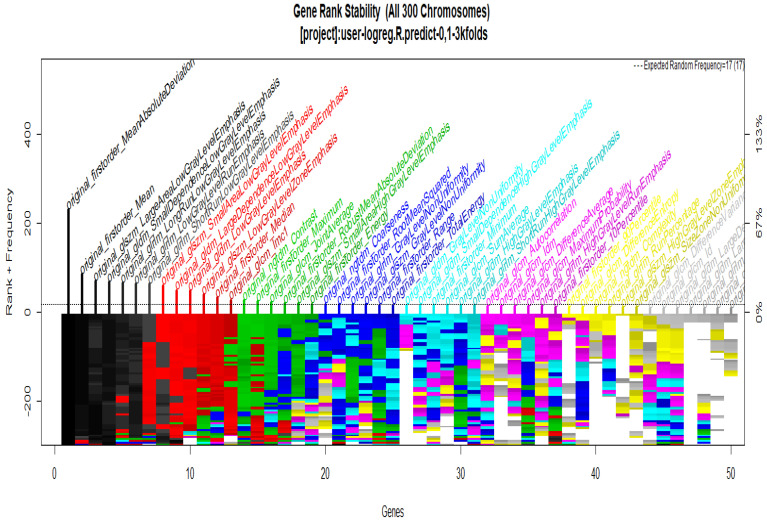
Gene Rank Stability graph with calcification model by radiologist segmentation; the gene rank shows the stability of the top-ranked 50 variables. The horizontal axis shows the genes ordered by rank, and the vertical axis shows the gene frequency. The bottom color—coded rank represents the genetic stability, where features with no change in color represent a stable feature; changing color features represent unstable features.

**Figure 13:**
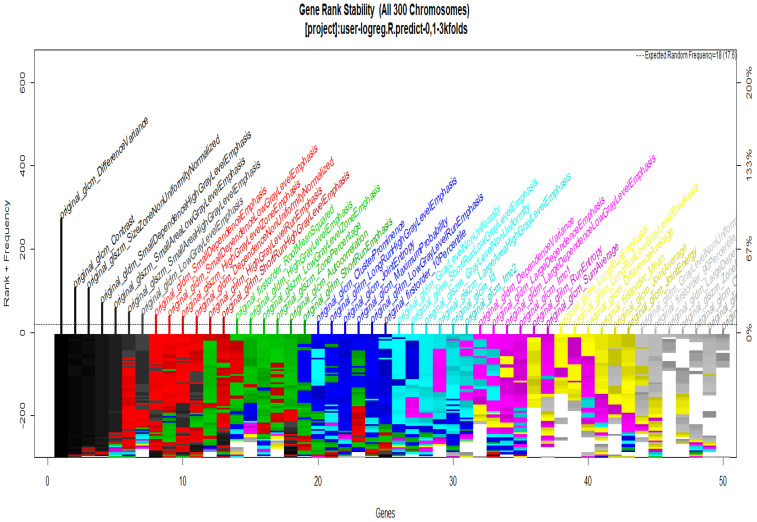
Gene Rank Stability graph with calcification model by radiologist segmentation; the gene rank shows the stability of the top-ranked 50 variables. The horizontal axis shows the genes ordered by rank, and the vertical axis shows the gene frequency. The bottom—coded rank represents the genetic stability, where features with no change in color represent a stable feature; changing color features represent unstable features.

To start with the feature extraction, the four groups CS1, CS2, MS1 and MS2 and their corresponding binary masks were selected as the input for the pyRadiomics system. The pyRadiomics process extracted 110 features; these features were related to the shape, and those with zero variance were removed, giving a grand total of 88 texture features. The GA (Galgo) algorithm analyzes different models obtained through evolution, with a maximum of 300 generations. The obtained models from the evolution process of the algorithm are shown in the Figure 10, Figure 11, Figure 12 and Figure 13. Horizontal axis genes ordered by rank and vertical axis shows the gene frequency and the colour-coded rank of each gene in previous evolutions. Changes in ranks are marked by different colours. These figures summarize the population of chromosomes within each generation, where the black color represents the most stable chromosome in all generated models.

In Figure 11 and Figure 13, seven black stable chromosomes were generated for ROI segmentation. However, for segmentation by the radiologist, as shown in Figure 10 and Figure 12, seven black stable chromosomes were obtained. Finally, Table 3 and Table 4 show a comparison of chromosomes generated in each model.

The global AUC criteria was also calculated by taking the average of all implemented models.

Table 5 shows a comparison between the results of the experiments with the CS1 and the CS2 data set.

The same comparison process as above is performed but now using the mass data set, as shown in Table 6. Features of black color represent the importance of predicting cancer.

In Table 7, the best predictors for the classification between benign or malignant using logistic regression for each CS1, CS2, MS1 and MS2 models are shown.

Moreover, to validate the results obtained with the proposed methodology, the accuracy and AUC results are compared with other proposals; the results are shown in Table 8.

## 5. Discussion

Results obtained when using the sub-sets CS1, CS2, MS1 and MS2 to classify calcification and masses were as good as it could be expected, which means, for example, that the obtained AUC was at least 0.8 for calcification and at least 0.9 for masses. The whole predictive measures obtained by the data set of calcification and mass between regions of interest are shown in Table 5 and Table 6. As shown, the predictive accuracy between the data set of CS1 is 86% and CS2 is 76%. The minimal difference is 12% according to the two models to predict malignant or benign images. On the other hand, for the results from Table 6, the predictive accuracy between the models MS1 is 95% and MS2 is 74%. In the comparison between the two previous models, the difference was 22% in accuracy. Finally, the evidence suggests that the prediction model CS2 (Calcification) has a higher probability of predicting the MS2 (Malignant) with a percentage of 10% of error.

According to Table 7, the results demonstrate that, for classification purposes, the measures of GLCM Difference Entropy, GLCM Contrast and GLCM Difference Variance are strongly correlated in the cases of CS1, MS1, and MS2 models. The relation between CS1 and MS2 models is given with NGTDM Business features. Finally, CS1 and MS1 models are correlated by GLCM Id and First Order Total Energy features. GLCM Difference Entropy is other measure of correlation that presents MS1 and MS2 cases. This experiment demonstrates that the GLCM class provides strong prediction measures to classify between malignant or benign class models. The most important result that emerges from the analysis in this section is the relationship between breast mass and cancer, and, respectively, between breast calcification and cancer; there are three radiomic features from the classes such as GLRLM, GLSZM, and GLCM, which are considered stable. Another advantage of the selection procedure used in the proposed methodology, is the dimensionality reduction with a 20% in the generation of a new optimal model.

The results provided in Table 8 give a comparison with respect to other state-of-the-art methodologies. In order to observe the veracity of the proposed methodology, some comparisons are made with respect to the other four methods, which employ the same database used in this research (CBIS_DDSM). It is important to say that these methods evaluate benign and malignant lesions according to calcification and mass mammograms images using two projections, MLO and CC. The obtained results with the proposed methodology outperform those results reported by [34,36], for benign and malignant lesions, for example for the MS1 case, and the area under the curve (AUC) given by our proposition is about 0.95 and 0.96 of accuracy. The AIC score (CS1, CS2, MS1 and MS2) is given for the MS2 model with 166—the lowest score as the best.

Feature extraction provides information for classifying breast lesions, and it is possible to make a good feature selection using logistic regression classification based on the texture image. This study found that the mass provides more information for classification, but the calcifications do not necessarily give more information. The calcifications could be segmented and, subsequently, features were extracted. The relationship between mass, calcification and cancer has the best classification rates when it is evaluated by the Gray Level Co-occurrence Matrix.

On the other hand, the image analysis performed by [35] also evaluates two types of lesions for calcification and masses with two projections, obtaining an AUC of 0.84 and 0.8 of accuracy. Moreover, in this comparison for the CS1 case, the proposed method gives a better result, since it obtained an AUC of 0.86 and 0.82 of accuracy. However, the AIC score (CS1, CS2, MS1 and MS2) is given for the MS2 model with 166—the lowest score as the best.

It has been demonstrated that the CC projection analysis provide the best information for the benign and malignant lesions classification, making an optimal feature extraction from the mammal tissue.

## 6. Conclusions

The detection of breast cancer at an early stage can be prevented from spreading to other parts of the body or avoiding death in the patient. The integration of predictive models in the diagnosis of breast cancer have allowed the radiologist to make quick decisions. Comparing a lesion breast analysis realized by a radiologist and the segmentation of the breast on mammography made by the classification models implemented in this work, there is no substantial difference in decision making. The implementation of genetic algorithms was considered in order to help to choose the best predictors in the detection of breast cancer; the results of the models implemented have a 86% AUC for calcification models and 95% of AUC for mass models.

Although there is much research focused mainly on finding the region of interest, this type of analysis would allow finding types of lesions in a very restricted area. In this new methodology, we propose an automated segmentation based on the analysis of the whole breast region to classify between benign and malignant lesions. The results demonstrate that between the lesion and the whole breast there is around a 10% of difference for cases of calcifications, and a 20% of difference in the case of masses. Based on the previous results, the radiologist would focus on the cases where the system finds malignant cases, and carry out a more in-depth study of the case. Our proposal allows us to speed up the work of the radiologist in decision-making.

The purpose of the present investigation is not to change the opinion of the radiologist, but to motivate the use of an alternative tool that allows one to improve the response time of the analysis in the detection of malignant or benign lesions in images with calcification or mass. The Pyradiomics system provided optimal features for a good classification. However, this system is limited by both the processing speed and the amount of memory available. 

## Figures and Tables

**Table 1:** DICOM information.

Information	Data
File Size	50,912,930 Bytes
Width	3796
Height	6706
BitDepth	16
ColorType	Grayscale
Categories	The abnormality from 1 to 5
Data patient	Age
FileMetaInformationVersion	uint8
Format	Tagged Image File Format (TIFF)
Type	Calcification or mass
Patology	Benign or malign
Views	MLO and CC

**Table 2:** Radiomics features for each class (See Appendix A).

Features	Number of Features
First Order statics	19
Gray Level Co-occurrence Matrix (GLCM) [31]	24
Gray level Run Length Matrix (GLRLM) [42,43]	16
Gray level Size Zone Matrix (GLSZM ) [44]	16
Neighbouring Gray Tone Difference Matrix (NGTDM)	5
Gray level Dependence Matrix (GLDM)	14
Total	94

**Table 3:** Comparison between models with CS1 and CS2 calcifications.

Measures	Radiologist Segmentation	Segmenting Breast Regions
1	First Order Mean Absolute Deviation	NGTDM Complexity
2	NGTDM Busyness	GLCM Diference Entropy
3	GLCM IMC2	GLRM Long Run High Gray Level Emphasis
4	GLDM Dependence Non Uniformity	GLCM idn
5	GLRM Run Length Non Uniformity	GLDM Small Dependence Emphasis
6	GLSZM Small Area Low Gray Level Emphasis	GLSZM Small Area High Gray Level Emphasis
7	GLCM IMC1	GLDM Dependence Non Uniformity Normalized

**Table 4:** Comparison between models with MS1 and MS2 mass.

Measures	Radiologist Segmentation	Segmenting Breast Regions
1	First Order Mean Absolute Deviation	GLCM Difference Variance
2	First Order Mean	GLCM Contrast
3	GLCZM Large Area Low Gray Level Emphasis	GLSZM Size Zone Non Uniformity Normalized
4	GLDM Small Dependence Low Gray Level Emphasis	GLDM Small Dependence High Gray Level Emphasis
5	GLRLM Run Long Run Low Gray Level Emphasis	GlSZM Small Area Low Gray Level Emphasis
6	GLRLM Low Gray Level Run Emphasis	GLSZM Small Area High Gray Level Emphasis
7	GLRLM Short Run Low Gray Level Emphasis	GLDM Low Gray Level Emphasis

**Table 5:** Comparison between models with CS1 and CS2 calcifications.

Measures	Radiologist Segmentation	Segmenting Breast Regions
AUC	0.86	0.73
Specificity	0.74	0.73
Sensitivity	0.91	0.66
Accuracy	0.82	0.77
AIC	178.68	180.83

**Table 6:** Comparison between models with MS1 and MS2.

Measures	Radiologist Segmentation	Segmenting Breast Regions
AUC	0.95	0.74
Specificity	1	0.78
Sensitivity	0.93	0.67
Accuracy	0.96	0.72
AIC	176.62	166

**Table 7:** The following table compares those features (in bold) that repeat in the different models (CS1, CS2, MS1 and MS2). These features provide better and more meaningful results for the classification of malignant cancer.

Calcification		Mass	
CS1	CS2	MS1	MS2
Feature	Feature	Feature	Feature
**NGTDM Busyness**	First Order Mean Absolute Deviation	NGTDM Complexity	**NGTDM Busyness**
**GLCM Difference Entropy**	First order Mean	**GLCM Difference Entropy**	**GLCM Difference Entropy**
GLCM Inverse Variance	-	GLCM Joint Energy	GLSZM Small Area Low Gray Level Emphasis
GLDM Dependence Non Uniformity	-	GLCM Difference Variance	GLSZM Low Gray Level Zone Emphasis
GLRLM Run Length Non Uniformity	-	GLRLM Run Percentage	
GLSZM Zone Variance	-	NGTDM Coarseness	GLDM Small Dependence Low Gray Level Emphasis
**GLCM Contrast**	-	**GLCM Contrast**	**GLCM Contrast**
**GLCM Difference Variance**	-	**GLCM Difference Variance**	**GLCM Difference Variance**
GLSZM Large Area High Gray Level Emphasis	-	GLSZM Low Gray Level Zone Emphasis	First Order Skewnees GLCM Contrast
**First Order Total Energy**	-	**First Order Total Energy**	GLSZM Gray Level Non Uniformity
	-	GLCM Id	GLDM Gray Level Non Uniformity
GLSZM Large Area Emphasis	-		GLRLM Run Entropy
GLSZM Gray Level Variance	-	GLSZM Large Area Low Gray Level Emphasis	GLRLM Gray Level Non Uniformity
GLCM Correlation	-	**GLCM Difference Average**	**GLCM Difference Average**
GLDM Gray Level Variance	-	GLDM Large Dependence Emphasis	GLCM Joint Entropy
GLSZM Small Area Emphasis	-	GLSZM Small Area Low Gray Level Emphasis	GLRLM Short Run Low Gray Level Emphasis
Shape Maximum2D Diameter Row	-		
**GLCM Id**	-	**GLCM Id**	
GLCM Joint Energy	-	First Order Energy	Sum Squares
GLRLM Gray Level Varariance	-	GLCM Maximum Probability	First Order Median
First Order Entropy	-	GLRLM Long Run Emphasis	GLDM Dependence Entropy

**Table 8:** CBIS-DDSM segmentation show the comparison of the results obtained using the methodologies proposed in [33,34,35,36].

Authors	Models	View	Data Set	Accuracy	AUC
Tsochatzidis et al. [33]	CNNs	CC and MLO	Mass	0.74	0.80
Andrik et al. [35]	DNN	CC and MLO	Calcification and Mass	0.80	0.84
Tsochatzidis et al. [34]	SVM	CC and MLO	Mass	0.81	0.85
Arora et al. [36]	Neural Network (nntraintool)	CC and MLO	Mass	0.88	0.88
**Our Method**	**CS1—Model**	**CC**	**Calcification**	**0.82**	**0.86**
**Our Method**	**MS1—Model**	**CC**	**Mass**	**0.96**	**0.95**
Chougrad et al. [37],	CCNs (Data set DDSM)	CC and MLO	Mass	0.97	0.98
Chougrad et al. [37]	CCNs (Data set INBreast)	CC and MLO	Mass	0.95	0.97
Chougrad et al. [37]	BCRDs (Data set BCRDs)	CC and MLO	Mass	0.96	0.96

## Data Availability

https://wiki.cancerimagingarchive.net/display/Public/CBIS-DDSM accessed on 22 May 2021.

## Abbreviations

The following abbreviations are used in this manuscript:

ROI	A Region Of Interest
ROC	Area under the curve
DDSM	The Digital Database for Screening Mammography
CBIS-DDSM	Curated Breast Imaging Subset of DDSM
DICOM	Digital Imaging and Communication In Medicine
IBSI	Imaging Biomarker Standardization Initiative
GA	Gentic Algorithm
AUC	Area under the ROC Curve
CC	Craniocaudal
LD	Linear dichroism
GLCM	A Gray Level Co-occurrence Matrix
GLRLM	Gray level Run Length Matrix
GLSZM	Gray level Size Zone Matrix
NGTDM	Neighbouring Gray Tone Difference Matrix
GLDM	Gray level Dependence Matrix
TIFF	Tagged Image File Format
CNNs	Deep Convolutional Neural Networks
DNN	Deep neural network
SVM	Support vector Machines
CNN	Convolutional Neural Networks

## Appendix A

**Table A1:** Radiomic Features.

Abbreviation for Feature	Definition
NGTDM Coarseness	Contrast Feature indicates the level of the spatial rate of change in intensity.
NGTDM Busyness	Feature describe the changes in the intensity between neighbouring pixels.
NGTDM Complexity	Features describe how are common are the non uniformity and rapid changes in the gray levels.
GLCM Energy	Angular second Momentum, Uniformity.
GLCM Contrast Variance	
GLCM Difference Entropy	Difference Average measures the relationship between occurrences of pairs with similar intensity values and occurrences of pairs with differing intensity values.
GLCM ID	Inverse difference normalizes the difference between the neighboring intensity values by dividing over the total number of discrete intensity values.
GLCM Maximum Probability	Maximum Probability is occurrences of the most predominant pair of neighboring intensity values.
GLCM Joint Entropy	Joint entropy is a measure of the randomness/variability in neighborhood intensity values.
GLCM Joint Energy	Energy is a measure of homogeneous patterns in the image
GLRLM Long Run Emphasis	LREis a measure of the distribution of long run lengths, with a greater value indicative of longer run lengths and more coarse structural textures.
GLRLM Gray Level Non-Uniformity	GLN measures the similarity of gray-level intensity values in the image.
GLRLM Gray Level Non-Uniformity Normalized (GLNN)	GLNN measures the similarity of gray-level intensity values in the image
GLRLM Run Length Non-Uniformity (RLN)	RLN measures the similarity of run lengths throughout the image.
GLRLM Run Length Non-Uniformity Normalized (RLNN)	RLNN measures the similarity of run lengths throughout the image.
GLRLM Run Percentage (RP)	RP measures the coarseness of the texture by taking the ratio of number of runs and number of voxels in the ROI.
GLRLM Gray Level Variance (GLV)	GLV measures the variance in gray level intensity for the runs.
GLRLM Run Entropy (RE)	RE measures the uncertainty/randomness in the distribution of run lengths and gray levels.
GLRLM Low Gray Level Run Emphasis (LGLRE)	HGLRE measures the distribution of the higher gray-level values.
GLRLM Short Run Low Gray Level Emphasis (SRLGLE)	RLGLE measures the joint distribution of shorter run lengths with lower gray-level values.
GLRLM Short Run High Gray Level Emphasis (SRHGLE)	measures the joint distribution of shorter run lengths with higher gray-level values.
GLRLM Long Run Low Gray Level Emphasis (LRLGLE)	measures the joint distribution of long run lengths with lower gray-level values.
GLRLM Long Run High Gray Level Emphasis (LRHGLE)	LRHGLRE measures the joint distribution of long run lengths with higher gray-level values.
NGTDM Coarseness	Coarseness is a measure of average difference between the center voxel and its neighbourhood and is an indication of the spatial rate of change.
NGTDM Contrast	Contrast is a measure of the spatial intensity change, but is also dependent on the overall gray level dynamic range.
NGTDM Busyness	Busyness a measure of the change from a pixel to its neighbour.
NGTDM Complexity	An image is considered complex when there are many primitive components in the image.
NGTDM Strength	Strength is a measure of the primitives in an image.
GLDM Small Dependence Emphasis (SDE)	A measure of the distribution of small dependencies, with a greater value indicative of smaller dependence and less homogeneous textures.
GLDM Large Dependence Emphasis (LDE)	Large Dependence Emphasis a measure of the distribution of large dependencies, with a greater value indicative of larger dependence and more homogeneous textures.
GLDM Gray Level Non-Uniformity (GLN)	Gray Level Non-Uniformity measures the similarity of gray-level intensity values in the image
